# Comparative Genomics Reveal Distinct Environment Preference and Functional Adaptation Among Lineages of Gemmatimonadota

**DOI:** 10.3390/microorganisms12112198

**Published:** 2024-10-31

**Authors:** Jiangtao Du, Zhixuan Wang, Lin Hu, Li Wang, Jiasong Fang, Rulong Liu

**Affiliations:** College of Oceanography and Ecological Science, Shanghai Ocean University, Shanghai 201306, China; 13127838459@163.com (J.D.); wangzhixuan1001@163.com (Z.W.); hulin9921@163.com (L.H.); l-wang@shou.edu.cn (L.W.); jsfang@shou.edu.cn (J.F.)

**Keywords:** Gemmatimonadota, functional profile, marine environment, genomic features

## Abstract

Bacteria in the phylum Gemmatimonadota are globally distributed and abundant in microbial communities of various environments, playing an important role in driving biogeochemical cycling on Earth. Although high diversities in taxonomic composition and metabolic capabilities have been reported, little is known about the environmental preferences and associated functional features that facilitate adaptation among different Gemmatimonadota lineages. This study systematically analyzed the relationships between the environments, taxonomy, and functions of Gemmatimonadota lineages, by using a comparative genomics approach based on 1356 Gemmatimonadota genomes (213 high-quality and non-redundant genomes) available in a public database (NCBI). The taxonomic analysis showed that the 99.5% of the genomes belong to the class Gemmatimonadetes, and the rest of the genomes belong to the class Glassbacteria. Functional profiling revealed clear environmental preference among different lineages of Gemmatimonadota, and a marine group and two non-marine groups were identified and tested to be significantly different in functional composition. Further annotation and statistical comparison revealed a large number of functional genes (e.g., *amiE*, *coxS*, *yfbK*) that were significantly enriched in genomes from the marine group, supporting enhanced capabilities in energy acquisition, genetic information regulation (e.g., DNA repair), electrolyte homeostasis, and growth rate control. These genomic features are important for their survival in the marine environment, which is oligotrophic, variable, and with high salinity. The findings enhanced our understanding of the metabolic processes and environmental adaptation of Gemmatimonadota, and further advanced the understanding of the interactions of microorganisms and their habitats.

## 1. Introduction

Bacteria in the phylum Gemmatimonadota are globally distributed and abundant in microbial communities across various environments [1,2], playing a significant role in driving biogeochemical cycling of biogenic elements such as carbon, nitrogen, and sulfur on Earth [1,2]. Environmental 16S rRNA gene sequence surveys indicate that they inhabit a wide range of ecosystems, including soil, polar regions, permafrost, rhizosphere, activated sludge, deep-sea sediments, freshwater lakes, brackish estuaries, natural gas hydrates, and marine sponge symbionts [3,4,5,6,7]. Gemmatimonadota rank among the eight most abundant phyla in soil, comprising 6.5% of the total 16S rRNA gene sequences in soils [7,8]. Additionally, Gemmatimonadota are prevalent in wastewater treatment, biofilms, and plant-associated environments [9]. Remarkably, Gemmatimonadota dominate the deepest oceanic trenches, such as the Mariana Trench and the Mussau Trench, where their average relative abundances in rRNA and rDNA libraries are 13.30 and 9.93%, respectively, indicating high potential activity among prokaryotic groups [10]. These studies underscore the extensive physiological diversity and significant ecological importance of Gemmatimonadota, highlighting their presence in a wide range of natural environments.

Gemmatimonadota are not only widely distributed but also exhibit highly diverse metabolic capabilities, contributing substantially to biogeochemical cycling [11]. Cultured strains of Gemmatimonadota have been shown to oxidize methane (CH_4_) and reduce nitrous oxide (N_2_O), thus playing a critical role in regulating greenhouse gas levels [12,13,14]. Furthermore, the abundance of Gemmatimonadota correlated positively with certain soil nutrients, indicating their key role in soil ecosystems [15]. In high-concentration urea wastewater treatment, Gemmatimonadota became the most abundant phylum associated with potential intracellular urea hydrolysis, utilizing urea as both an energy source and a crucial substrate [16]. Cultured Gemmatimonadota also showed antibiotic resistance that was enabling their growth in the presence of ampicillin or penicillin, as well as bacitracin and chloramphenicol [17,18,19]. Gemmatimonadota were also among the few phyla capable of anoxygenic photosynthesis, harnessing photosynthesis for additional energy acquisition [11,20]. Systematic analyses of existing Gemmatimonadota genomes (all belonging to the class Gemmatimonadetes) indicated that these bacteria primarily engaged in heterotrophic metabolism, capable of degrading various complex organic substrates [2], thus playing vital roles in marine carbon cycling and other biogeochemical processes [10].

In line with their wide distribution and diverse metabolic capabilities, Gemmatimonadota also exhibit high species diversity. According to the SILVA classification (Version 1.1.11/138.1 SSU Ref NR) [21], the phylum Gemmatimonadota comprises seven classes. However, only the classes Gemmatimonadetes and Longimicrobia have species that have been cultivated in laboratory settings [9], and the majority of 16S rRNA gene sequences are derived from uncultured species [22]. Gemmatimonadota from different habitats may exhibit significant differences in morphology, function, and lifestyle. For example, Gemmatimonadota in the lower layers of freshwater lakes are small, free-living cells, whereas those in the epilimnion are larger and associate with diatoms and cyanobacteria [3]. These adaptive traits may be reflected in their genomes. Indeed, pangenomic studies based on existing genomes reveal that Gemmatimonadota genomes possess high expansibility, likely due to their adaptation to diverse environments [2]. An extensive multi-environment analysis of publicly available Gemmatimonadota genomes up to May 2021 further demonstrated environment-specific features in gene composition (presence/absence) and general genomic characteristics, such as genome size and the number of coding sequences [11]. However, with the great increase in Gemmatimonadota genomes in public databases (e.g., NCBI), the environmental preference of these lineages needs to be revisited. In addition, the key genes and functions that facilitate their adaptation to different environments are still not clear.

In this study, we systematically analyzed existing Gemmatimonadota genomes and their environmental sources from public databases, elucidating the environmental preferences in the phylogeny and genome functional profiles of Gemmatimonadota. The bacteria of the class Gemmatimonadetes occupy the majority of known Gemmatimonadota genomes, exhibiting significant differences in functions and species composition between marine and non-marine groups. Functional metabolic differences further revealed the adaptive mechanisms of Gemmatimonadota to marine and non-marine environments. These findings provide a foundation for further understanding the ecological functions of Gemmatimonadota and the internal mechanisms of bacterial adaptation to different environmental conditions.

## 2. Materials and Methods

### 2.1. Data Acquisition

The genomes of Gemmatimonadota were downloaded from the NCBI database (14 August 2023). Briefly, all of the Gemmatimonadota genomes publicly available were searched on the website (https://www.ncbi.nlm.nih.gov/genome/browse#!/prokaryotes, accessed on 14 August 2023) using the term “Gemmatimonadota”, and the assembly options “Chromosome”, “Complete Genome”, “Scaffold”, and “Contig” were selected. The genomes were then downloaded using BioSAK (v1.73.5, --dwnld_GenBank_genome), which generated a dataset containing 1356 genomes.

### 2.2. Genome Dereplication and Taxonomic and Phylogenomic Analysis

The downloaded Gemmatimonadota genomes were dereplicated using dRep [23] (v2021.4.99, -pa 0.9-sa 0.95-comp 90-con 5). Taxonomic identification of the dereplicated Gemmatimonadota genomes was performed using GTDB-Tk [24] (v2.1.1, GTDB Release-R207), and genomes not belonging to the phylum Gemmatimonadota were removed. Completeness and contamination were assessed using CheckM [25] (v1.1.2), and only the genomes > 95% completeness and <5% contamination were retained. After the above processes, 213 high-quality and taxonomically confirmed Gemmatimonadota genomes were selected for downstream analysis, and the information on the environmental source of each Gemmatimonadota genome was retrieved from the NCBI database. A maximum likelihood phylogenomic tree was constructed using TreeSAK [26] (v1.28.0, -GTDB_tree) and tree visualization was performed using R.

### 2.3. Functional Annotation and Metabolic Reconstruction

Open reading frames (ORFs) within the non-redundant genomes were predicted using Prodigal [27] (v2.6.3) with default parameters. All ORFs were annotated using the KEGG (Kyoto Encyclopedia of Genes and Genomes) database [28] with GhostKOALA [29]. The generated gene profiles (gene list and their copy numbers) of the genomes were utilized for downstream comparison.

### 2.4. Comparison on Functional Profiles

The gene profiles (gene list and their copy numbers) from KEGG annotation were utilized for comparison of functional compositions between different genomes. Briefly, standardized gene profiles (gene copy number normalized by total copy number of the profile) were pair-wise-compared and a Bray–Curtis similarity matrix was generated using PRIMER 6 [30] (v6.1.10; PRIMER-E, Ivybridge, UK). Hierarchical clustering analysis and nonparametric multidimensional scaling (nMDS) were conducted using PRIMER 6 to show the differences between genomes from different taxonomies or different environment sources. One-way analysis of similarity (ANOSIM) was performed to test the differences between clusters or groups.

### 2.5. Identification of Functional Genes Related to Adaptation to Different Environments

The gene profiles of different groups were further compared and visualized with the Statistical Analysis of Meta-genomic Profiles (STAMP) v2.1.3 software package [31], and the genes with significant differences (*p*-value < 0.05, effect size > 0.1) between the compared groups were further identified using Welch’s *t*-test.

## 3. Results and Discussion

### 3.1. Biogeography and Taxonomy Analysis of Gemmatimonadota

A total of 1356 genomes of Gemmatimonadota available in the NCBI database were downloaded. After quality control (check M), dereplication (dRep [23], 95% ANI), and taxonomic confirmation (GTDB-TK [24]), 213 high-quality and non-redundant Gemmatimonadota genomes were retained (Table S1). These genomes were all with ≥95% completeness and ≤5% contamination (Table S1), and were retrieved from various habitats worldwide, including freshwater/terrestrial sediments (25.8%), marine animals (18.7%), soil (18.3%), activated sludge/sewage (11.7%), seawater/marine sediments (8.9%), alkaline salt/hypersaline lake (7.9%), cold seeps/hydrothermal vents (1.4%) and other environments (4.2%, including biofilm, fossil, wood decay, bark surface of an *Acer pictum* subsp, rock and aquaculture biofloc) (Figure S1). Genome sizes range from 1.82 to 7.48 Mb, with a median of 3.71 Mb, and their GC content ranges from 57.90% to 69.10%, with a median of 64.90% (Table S1). The variations in genome sizes and GC contents indicate high diversity within the phylum Gemmatimonadota.

**Figure 1:**
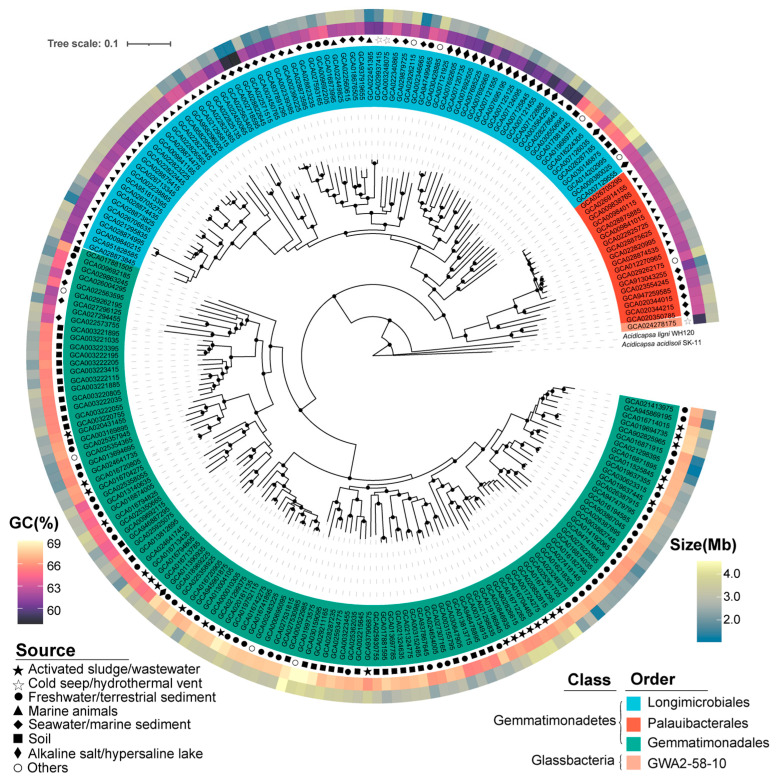
Maximum likelihood phylogenomic tree constructed from 213 selected Gemmatimonadota genomes with the genomes of Acidobacteria (GCA_025685655.1 GCA_025685625.1) used as the root of the tree. Bootstrap values were calculated with 100 replicates and values above 90% are indicated at the corresponding branch nodes. Different orders of Gemmatimonadota are marked with different colors. The inner middle and outer circles around the tree represent the environment sources’ GC content (%) and genome size (Mb), respectively. Detailed information about the genomes can be found in Table S1.

Based on phylogenomic analysis and GTDB taxonomies, the 213 representative Gemmatimonadota genomes were classified into four orders, namely Gemmatimonadales (118 genomes), Palauibacterales (18 genomes), Longimicrobiales (76 genomes), and GWA2–58–10 (1 genome) (Figure 1; Table S1). These orders belong to two classes, i.e., Gemmatimonadetes (212 genomes, 99.5% of the total genomes) and Glassbacteria (1 genome) (Figure 1; Table S1). The results were consistent with previous studies, which showed that the majority of the currently available Gemmatimonadota genomes belong to the class Gemmatimonadetes [2,11]. In contrast to earlier studies, which included genomes with highly varied qualities (completeness from 50% to 100%) [2,11], we only retained high-quality genomes (completeness > 95%) in our study to enable a more a comprehensive comparison. Comparison of the genomes revealed differences in genomic features and environmental preference between different taxa. Within the class Gemmatimonadetes, genomes of the order Longimicrobiales were phylogenomically close to Palauibacterales, and both were distinct from the order Gemmatimonadales (Figure 1). Most Longimicrobiales and Palauibacterales genomes were retrieved from marine environments or salt lakes, while Gemmatimonadales genomes were predominantly sourced from non-marine environments (Figure 1).

### 3.2. Environmental Preference of Gemmatimonadota Based on Functional Profiles

To explore whether different types of Gemmatimonadota exhibit habitat preferences in terms of metabolic functions, we performed cluster analysis based on the similarities between annotated gene profiles (gene list and their copy number proportion) of the Gemmatimonadota genomes (Table S2).

**Figure 2:**
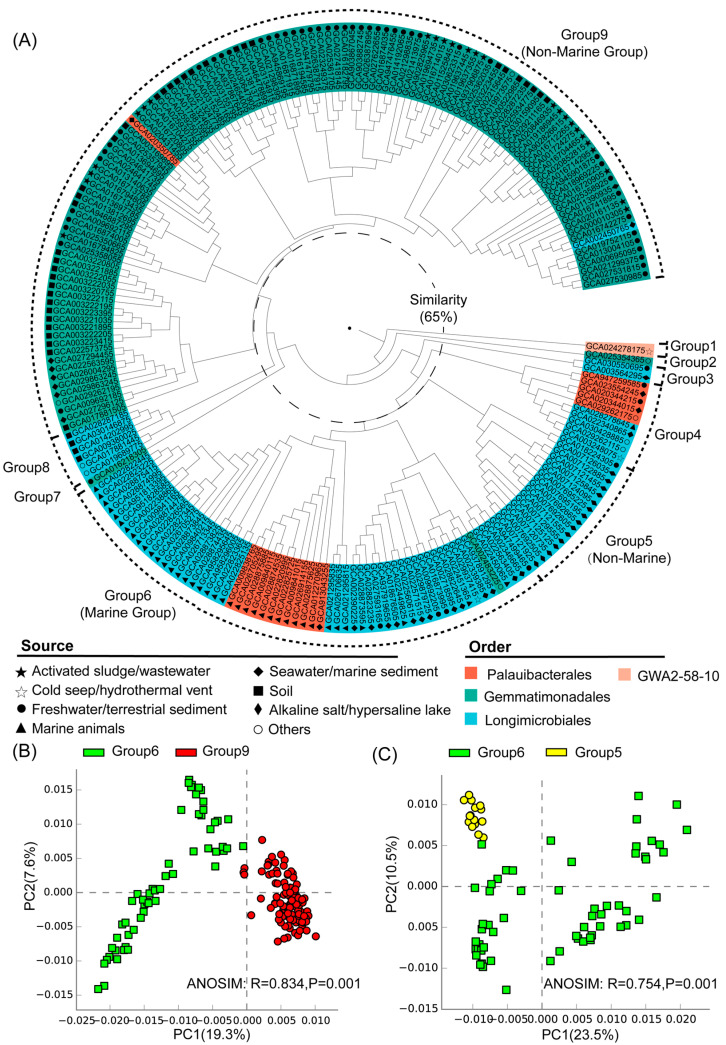
(**A**) Hierarchical clustering of Gemmatimonadota genomes based on Bray–Curtis similarities between annotated gene profiles (gene list and their copy numbers detailed in Table S2). The inner middle and outer circles around the dendrogram represent order-level taxonomies and their environmental sources, and the grouping of the Gemmatimonadota genomes, respectively. (**B**) PCA analysis for the marine (group 6) and non-marine groups (group 9) with statistical tests (ANOSIM). (**C**) PCA analysis for the marine (group 6) and non-marine groups (group 5) with statistical tests (ANOSIM).

Nine distinct groups were identified at a 65% similarity level, and most of these groups exhibited clear habitat preferences (Figure 2A). Group 1 corresponded to the class Glassbacteria (order GWA2–58–10), which was from the cold seep (Figure 2A). The genomes of groups 2–9 all belonged to the class Gemmatimonadetes, including the orders Gemmatimonadales, Longimicrobiales, and Palauibacterales (Figure 2A). Among them, groups 5, 6, and 9 were the largest clusters and they contained the majority of the analyzed genomes. Group 6 was predominantly (90%) composed of genomes from marine environments like seawater/marine sediments and marine organisms, and was therefore defined as the marine group. Taxonomically, the genomes of group 6 mainly belonged to the orders Longimicrobiales and Palauibacterales (Figure 2A). In contrast, group 9 was primarily (94%) from non-marine environments such as activated sludge/sewage, freshwater/terrestrial sediments, and soil, and was therefore defined as a non-marine group (Figure 2A). Moreover, group 5 was composed of genomes from alkaline salt lakes and hypersaline lakes, and was therefore defined as another non-marine group. Among the two non-marine groups, group 9 was mainly composed of genomes from the order Gemmatimonadales, while group 5 was composed of genomes from the order Longimicrobiales, which was close to the marine group (group 6) in hierarchical clustering. ANOSIM analysis further showed the statistically significant differences between the identified marine and non-marine groups (Figure 2, Figure S2), suggesting the existence of environment-specific genomic features.

### 3.3. Statistical Comparison of Genomic and Functional Features Related to Adaptations to Marine Environment

**Figure 3:**
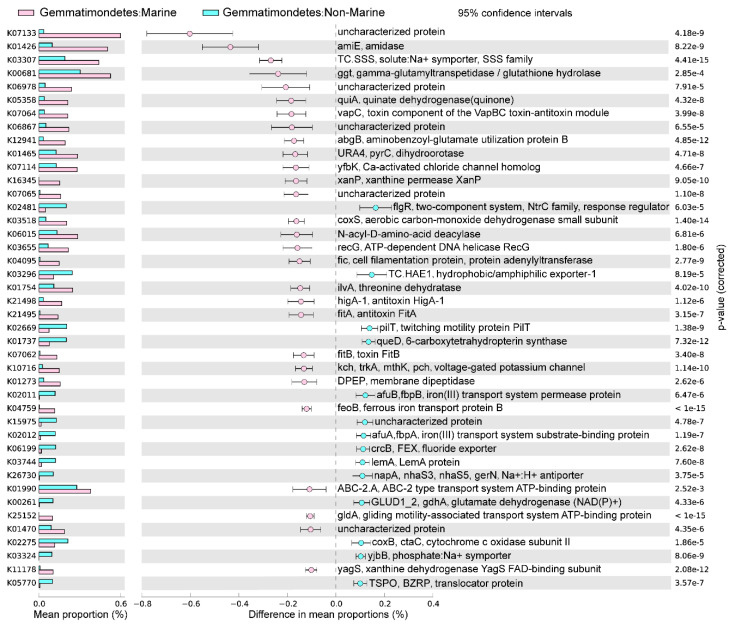
The differences in metabolism between marine (group 6) and non-marine groups (group 5). The statistical test method used was Welch′s *t*-test, and the confidence interval method was DP: Welch′s inverted, with a 95% confidence interval. The factors with effect values greater than 0.1 are selected and arranged in descending order.

**Figure 4:**
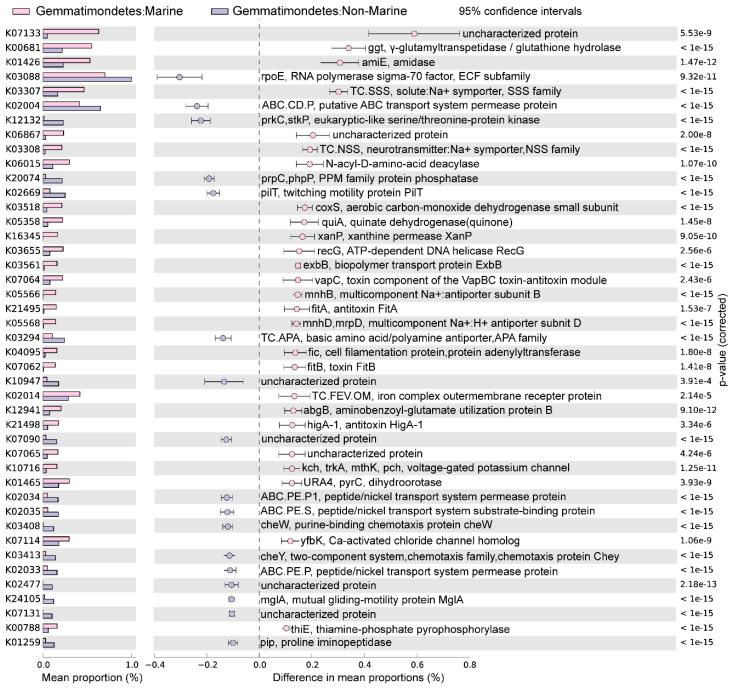
The differences in metabolism between marine (group 6) and non-marine groups (group 9). The statistical test method used was Welch′s *t*-test, and the confidence interval method was DP: Welch′s inverted, with a 95% confidence interval. The factors with effect values greater than 0.1 are selected and arranged in descending order.

**Figure 5:**
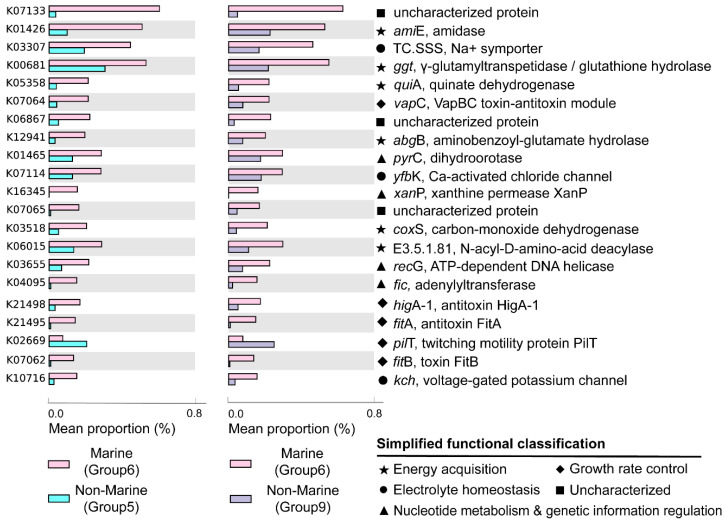
Genes significantly enriched in marine (group 6) or non-marine groups (group 5 and group 9). Only the KOs that were consistently identified in both the comparisons of group 6 vs. 5 and group 6 vs. 9 were shown. Factors were ranked by effect size in descending order and only those with an effect size greater than 0.1 were selected. Mean proportion means the average proportion of each corresponding gene in each genome. The gene names, their encoded enzymes, and the functions of enzymes are labeled at the right-hand side of the figure. The functional classifications of the genes were indicated with different symbols.

The marine (group 6) and non-marine groups (group 5 and 9) involved the majority of the currently available Gemmatimonadota genomes (99.1% of the 213 genomes) (Figure 2). Significant differences existed between the gene profiles of group 6 and group 5 (Figure 2C), as well as between those of group 6 and group 9 (Figure 2B), representing the metabolic differences between Gemmatimonadota in marine and non-marine environments. By statistically comparing the gene profile of the genomes from the marine group (group 6) and the two non-marine group (groups 5 and 9) using STAMP, the genes with effect sizes greater than 0.1 between these groups were identified (Figure 3 and Figure 4). The genes that were consistently enriched in the marine group in both the comparisons of group 6 vs. 5 and group 6 vs. 9 were further identified as functional genes that might be related to the adaptation to the marine environments (Figure 5). The results showed that genes enriched in genomes from marine environments were mainly related to energy acquisition, nucleotide metabolism and genetic information regulation, electrolyte homeostasis, and growth rate control (Figure 5; Table S3).

Most of the genes enriched in the genomes of the marine group encode enzymes participating in pathways for energy acquisition, including those for carbon, nitrogen, and sulfur compound metabolism (Figure 5, Table S3). Gemmatimonadetes genomes from marine environments have higher levels of *ggt* gene, which encodes γ-glutamyl transpeptidase/glutathione hydrolase (Figure 5). The enzyme catalyzes the hydrolysis of γ-glutamyl compounds such as glutathione (GSH) and the transfer of γ-glutamyl groups to amino acids and peptides [32]. As GSH is the most abundant thiol compound in cells, its hydrolyzation can serve as an important sulfur source for bacteria [32]. The gene *coxS* encoding carbon monoxide dehydrogenase was also significantly more abundant in marine groups (Figure 5). Carbon monoxide dehydrogenase catalyzes the reversible oxidation of carbon monoxide to carbon dioxide, allowing the organisms to utilize CO as source of carbon and energy [33]. It has been shown that carbon monoxide serves as a major energy source for the persistence of aerobic heterotrophic bacteria in nutrient-poor or variable environments [34]. In addition, Gemmatimonadetes genomes from marine environments have higher levels of *abgB*, *amiE*, and E3.5.1.81 genes, encoding p-aminobenzoyl-glutamate hydrolase, amidase, and N-acyl-D-amino acid deacylase, respectively (Figure 5). All of the three enzymes were hydrolases acting on amide bonds [35,36,37], a process that breaks down amides into their corresponding acids and amines. The amine compounds produced from amide hydrolysis can serve as a nitrogen source, and the generated organic acids can be utilized as a carbon source for microorganisms [38,39]. Hence, the hydrolysis of amide bonds plays vital roles in carbon and nitrogen metabolism in microorganisms [40]. Overall, by enhancing the capability for the hydrolysis of glutamyl compounds and amides, or the oxidation of carbon monoxide, Gemmatimonadetes might be able to acquire energy from diverse substrates to support their survival in the ocean. However, the roles of these genes in the adaptation of the marine group Gemmatimonadetes need to be further explored.

Moreover, Gemmatimonadetes genomes from marine environments have higher levels of *fic*, *recG*, *xanP*, and *pyrC* genes, encoding adenylyltransferase, ATP-dependent DNA helicase, and xanthine permease, respectively (Figure 5). These enzymes were involved in nucleotide metabolism and genetic information processing: the ATP-dependent DNA helicase encoded by *recG* can promote DNA unwinding [41], while the adenylyltransferase encoded by the *fic* gene can catalyze the addition of adenosine monophosphate (AMP) to Rho GTPases, preventing their interaction with downstream effectors and thereby inactivating them [42,43]. The xanthine permease encoded by *xanP* is involved in the transfer of xanthine, which is a purine formed during the catabolism of guanine [44]. The gene *pyrC* encodes dihydroorotase, which is involved in pyrimidine metabolism and cofactor biosynthesis within the cell [45]. Enhanced helicase activity in microorganisms allows more rapid and efficient DNA repair, thereby reducing gene mutations [46]. Additionally, many marine microorganisms acquire new genes through horizontal gene transfer [47], and helicases aid in the integration of foreign DNA, which promotes genetic diversity and adaptive evolution [48]. However, due to the lack of information about gene mutation and horizontal gene transfer in marine Gemmatimonadetes, the roles of these genes in aiding the adaption of the bacteria to marine environments need to be further studied.

The genomes of the marine groups showed enrichment of the *mnhB* gene encoding Na+ transport proteins (TC.SSS) [49] (Figure 5). These proteins drive Na+ coupling, flagellar rotation, pH regulation, and cell volume regulation in alkaline environments [50]. The genomes of the marine group also harbored a higher level of genes encoding voltage-gated potassium channels (*Kch*, *TrkA*, *MthK*, *Pch*) (Figure 5), which are prokaryotic potassium channels that can be selectively activated by Ca^2+^, Mg^2+^, Mn^2+^, and Ni^2+^ [51]. These channels are central to various biological processes, including electrical signal transduction, electrolyte homeostasis, and cell volume regulation [51]. The higher level of genes encoding proteins related to the transportation of different ions is important for Gemmatimonadota for adaptation to the salty conditions of the marine environment.

The genomes of marine Gemmatimonadota also showed elevated levels of genes *higA−1*, *fitB*, *fitA*, and *vapC* (Figure 5), which were associated with the production of toxins and antitoxins involved in growth rate regulation. *fitA* is likely a DNA-binding protein that may regulate cell replication [52]. *fitB* is a potent toxin that can inhibit bacterial growth, while *higA* is an antitoxin that counteracts *fitB*′s toxicity by co-expression or delayed production, thereby preventing *fitB*-induced growth inhibition [53]. Additionally, *vapC*, which encodes a magnesium-dependent ribonuclease, plays a crucial role in regulating growth rates and toxicity among different bacterial species [54]. The product of *vapC* can display toxicity in vivo under conditional expression and plays an important role in switching between rapid and slow growth rates of bacteria [54]. The observed capacity for self-regulation of cellular growth may be a crucial feature of the adaptation of Gemmatimondota to the challenging conditions of marine environments, as Gemmatimondota have been speculated to maintain their metabolic activity and resistance to environmental stresses by sustaining a low growth rate [9].

## 4. Conclusions

In this study, we conducted a comprehensive analysis on the environmental preference and adaptive features of the phylum Gemmatimonadota by examining publicly available high-quality, non-redundant genomes from a variety of global habitats. Phylogenomic and functional profiling of these genomes revealed clear habitat preferences among different lineages of Gemmatimonadota. Specifically, genomes from marine environments primarily belonged to the orders Longimicrobiales and Palauibacterales, and exhibited increased gene contents for energy acquisition, nucleotide metabolism, ion transport, and growth regulation. These adaptations likely enable their survival in the oligotrophic and variable conditions of marine habitats. Overall, the findings underscore the remarkable functional diversity within the phylum Gemmatimonadota and highlight the ecological significance of its adaptations to various environments. The results from this study can serve as valuable references for generating hypotheses and validating experiments related to the adaptation mechanisms of microorganisms. However, the currently available genomes of Gemmatimonadota are primarily restricted to a limited number of taxa (e.g., the class Gemmatimonadetes) and environments. Further exploration of Gemmatimonadota, particularly from the under-represented taxa and habitats, is crucial for uncovering the full extent of their metabolic versatility and evolutionary history.

## Data Availability

All supporting data, code, and protocols have been provided within the article or through supplementary data files. Two supplementary figures and three supplementary tables are available in the online version of this article. The sources and genomic sequences used throughout this study have been deposited in the National Centre for Biotechnology Information (NCBI), under the assembly accession numbers provided in Table S1 (available in the online version of this article).

## Supplementary Materials

The following supporting information can be downloaded at: https://www.mdpi.com/article/10.3390/microorganisms12112198/s1, Figure S1: Global distribution of genome and proportion of different environments; Figure S2: PCA analysis of non-marine (Group 5 and Group 9) and marine (Group 6) groups; Table S1: Original genomic information; Table S2: KEGG annotation results and gene copy number; Table S3: Functions from the literature and simplified functional classification.
