# Supplementary material for: Comparative Genomics Reveal Distinct Environment Preference and Functional Adaptation Among Lineages of Gemmatimonadota
Source: Microorganisms. 2024 Oct 31;12(11):2198. doi: 10.3390/microorganisms12112198 (PMC11596202; doi:10.3390/microorganisms12112198)
Supplement: Supplementary file 1 [file microorganisms-12-02198-s001.zip › Supplementary Figures.pdf]

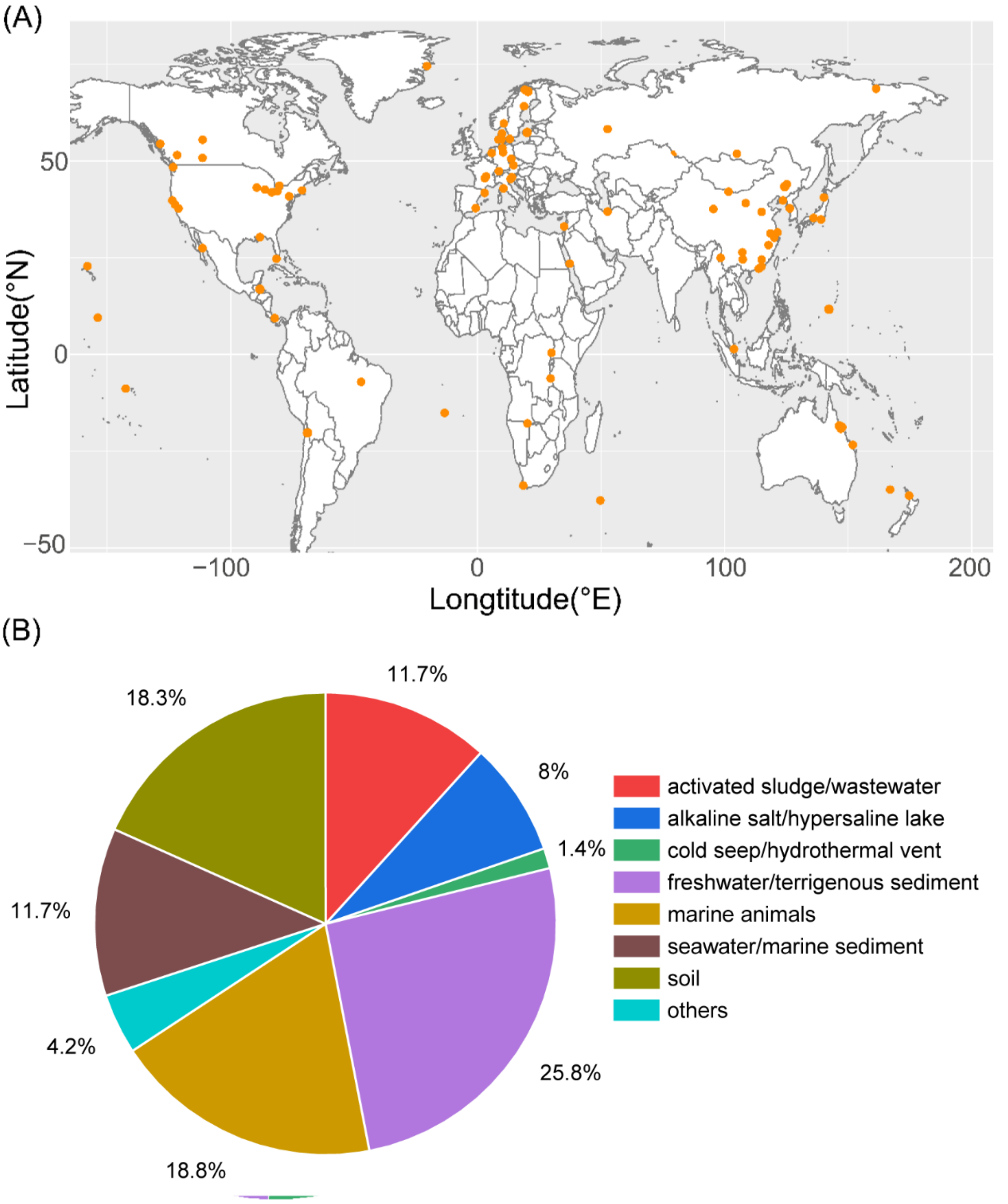

Figure S1. (A) Environment sources of the 213 representative Gemmatimonadota genomes analyzed in this study. (B) Proportion of Gemmatimonadota genomes from different environments.

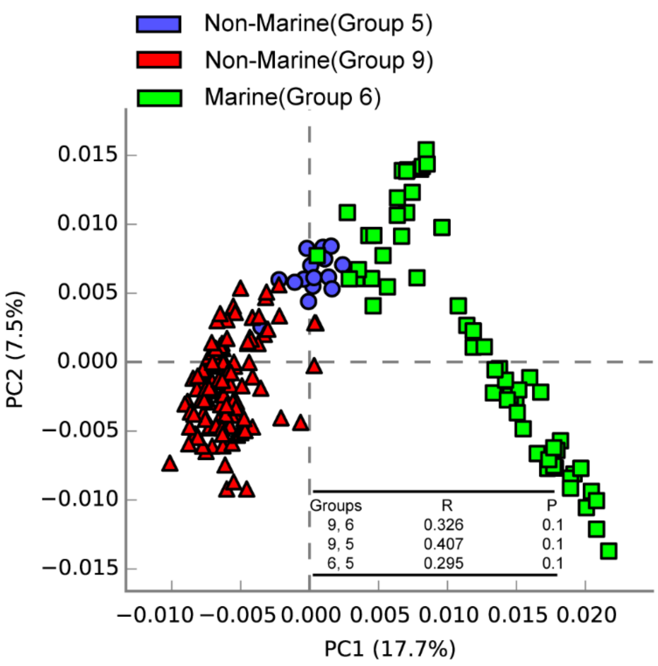

Figure S2. PCA analysis of non-marine (Group 5 and Group 9) and marine (Group 6) groups. The R and P values of the ANOSIM analysis results between the three groups are labeled in the figure.
